# *In Vivo* Characterisation of Five Strains of Bovine Viral Diarrhoea Virus 1 (Subgenotype 1c)

**DOI:** 10.3390/pathogens7010012

**Published:** 2018-01-19

**Authors:** Rebecca K. Ambrose, Jennifer L. Gravel, Margaret A. Commins, Elizabeth V. Fowler, Timothy J. Mahony

**Affiliations:** 1 Department of Agriculture and Fisheries, Animal Science, Dutton Park 4102, Australia; rebecca.ambrose@daf.qld.gov.au (R.K.A.); jenny.gravel@daf.qld.gov.au (J.L.G.); margaret.commins@daf.qld.gov.au (M.A.C.); elizabeth.fowler@daf.qld.gov.au (E.V.F.); 2 Queensland Alliance for Agriculture and Food Innovation, The University of Queensland, St Lucia 4072, Australia

**Keywords:** bovine viral diarrhoea virus 1, BVDV-1, subgenotype 1c, in vivo properties, infection, pyrexia, leukopenia

## Abstract

Bovine viral diarrhoea virus 1 (BVDV-1) is strongly associated with several important diseases of cattle, such as bovine respiratory disease, diarrhoea and haemoragic lesions. To date many subgenotypes have been reported for BVDV-1, currently ranging from subgenotype 1a to subgenotype 1u. While BVDV-1 has a world-wide distribution, the subgenotypes have a more restricted geographical distribution. As an example, BVDV-1 subgenotypes 1a and 1b are frequently detected in North America and Europe, while the subgenotype 1c is rarely detected. In contrast, BVDV-1 subgenotype 1c is by far the most commonly reported in Australia. Despite this, uneven distribution of the biological importance of the subgenotypes remains unclear. The aim of this study was to characterise the in vivo properties of five strains of BVDV-1 subgenotype 1c in cattle infection studies. No overt respiratory signs were reported in any of the infected cattle regardless of strain. Consistent with other subgenotypes, transient pyrexia and leukopenia were commonly identified, while thrombocytopenia was not. The quantity of virus detected in the nasal secretions of transiently infected animals suggested the likelihood of horizontal transmission was very low. Further studies are required to fully understand the variability and importance of the BVDV-1 subgenotype 1c.

## 1. Introduction

Bovine respiratory disease (BRD) is the most important disease of intensively finished cattle. While multiple factors contribute to the likelihood of cattle developing BRD, a generally accepted model for BRD development is a primary viral infection predisposing cattle to more severe secondary bacterial infections. Several viruses have been associated with an increased risk of BRD, including bovine herpesvirus 1 (BoHV-1), bovine viral diarrhoea virus 1 (BVDV-1), bovine respiratory syncytial virus and bovine parainfluenza 3 virus.

Of the key BRD associated viruses, BVDV-1 is the most genetically diverse with 21 subgenotypes, BVDV-1a to BVDV-1u, having been reported [1]. Yesilbag et al. [1] recently reviewed the geographical distribution of the BVDV-1 subgenotypes. This analysis highlighted several unusual trends in the distribution of the BVDV-1 subgenotypes. As an example, the vast majority of genotyped strains identified in Australia have been classified within the subgenotype 1c [2,3]. This is in contrast to the USA where the subgenotype 1b is dominant, but the subgenotype 1a is also frequently reported [4]. While in Europe the most common subgenotypes are 1a and 1b, also common are subgenotypes 1d, 1e, 1f and 1h [1]. However, the distribution of subgenotypes can vary between countries within Europe, for example subgenotype 1b and 1d were recently reported to be the most frequently identified subgenotypes in Germany [5].

Strains of the subgenotype 1c have been rarely reported in the USA or Europe. The drivers of these distributions are unclear, but do not appear to be influenced by vaccine use [6]. Several studies have suggested that the subgenotypes, at least in part, also reflect the antigen diversity between strains using cross-neutralisation assays [3,7,8]. Clearly the degree of cross-protection afforded by the subgenotype(s) included in a BVDV-1 in a vaccine against the subgenotypes circulating in a cattle population is an important issue.

The overall biological importance of the BVDV-1 subgenotypes remains to be fully elucidated since the majority of published studies have focused on the BVDV-1a and BVDV-1b genotypes. It is reasonable to accept that the subgenotypes of BVDV-1 share similar properties with respect to their capacity to infect susceptible ruminants, and their contribution to the development of more severe clinical disease outcomes such as BRD and the birth of persistently infected calves following transplacental infection. All of these outcomes are reported in cattle populations regardless of what subgenotype is present.

The evaluation of any variation of the in vivo properties of BVDV-1 subgenotypes has received little attention. To assist in developing a better understanding of the importance of the BVDV-1 subgenotypes, the aim of this study was to assess the in vivo properties of five BVDV-1 strains from the poorly studied subgenotype 1c. 

## 2. Results

### 2.1. Clinical Assessments

No overt clinical scores were recorded for any of the trial animals, challenged or unchallenged with the BVDV-1c strains used in this study (data not shown).

### 2.2. Rectal Temperatures

**Figure 1:**
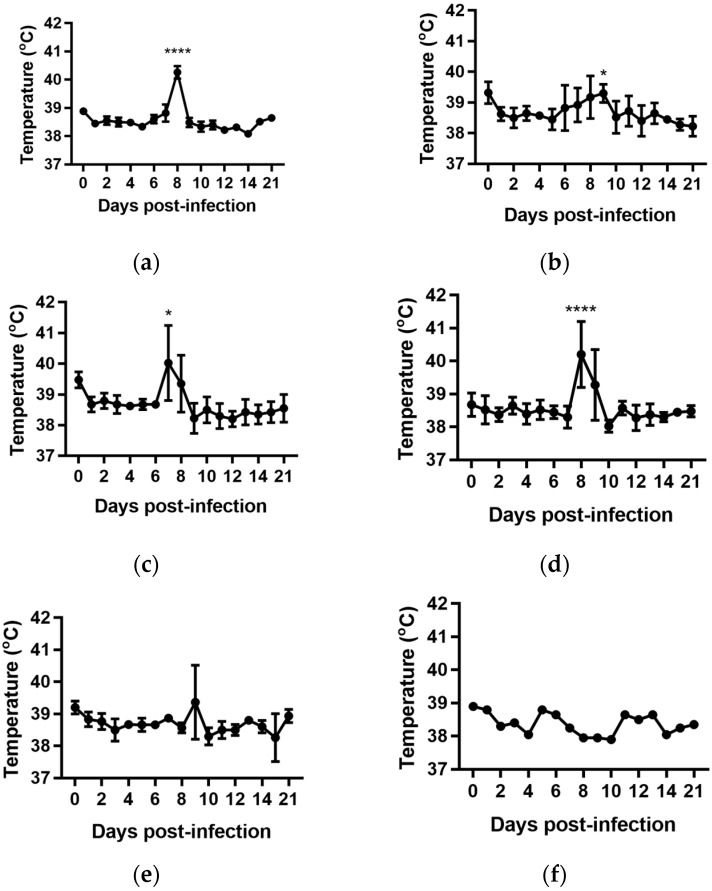
Rectal temperatures from groups of cattle infected with different strains of bovine viral diarrhoea virus 1 genotype 1c (BVDV-1c). The mean temperature with the standard error of the mean are shown for each day post infection. (**a**) Animals infected with BVDV-1c strain PI506 (*n* = 6); (**b**) Animals infected with BVDV-1c strain Trangie (*n* = 4); (**c**) Animals infected with BVDV-1c strain AO554 (*n* = 4); (**d**) Animals infected with BVDV-1c strain NS155 (*n* = 4); (**e**) Animals infected with BVDV-1c strain VR1112 (*n* = 3); (**f**) Uninfected animals (*n* = 2). The asterisks above selected days indicate significant differences compared to Day 1 post-infection for that group of cattle. Level of significance; * *p* < 0.05; **** *p* < 0.0001.

For all groups, except the cattle infected with BVDV-1c strain NS155, there was a general trend for an increase in the average rectal temperature on Day 0 of the experiment, prior to any viral exposure, compared to the temperatures from Day 1 to Day 5 (Figure 1a–f). The reason(s) for this increase are unclear. The cattle had been inducted into the containment facility seven days prior to the commencement of the trial and were therefore considered to have been well adapted/acclimatised to the environment. However, on Day 0 there were extra staff and equipment present while the cattle were being inoculated which included extra handling of the animals/cattle potentially contributing to this apparent increase. Due to this anomaly, the Day 1 temperatures were used as the reference point in the subsequent statistical comparisons within each treatment group.

The daily rectal temperatures were monitored for the trial cattle for 14 days following experimental infection. Overall the mean rectal temperature results were variable (Figure 1). Animals (*n* = 6) infected with BVDV-1c strain PI506 exhibited a significant temperature elevation on Day 8 post infection (*p* < 0.001, Figure 1a). For animals infected with BVDV-1c strain Trangie (*n* = 4), the temperature increase was gradual from Day 6 to Day 9 post infection, although the increase was only statistically significant on Day 9 (*p* < 0.05, Figure 1b). Animals infected with BVDV-1c strain AO554 (*n* = 4) exhibited elevated mean temperatures on Day 7 and Day 8 post infection, with only the Day 8 mean temperature being statistically significant (*p* < 0.05, Figure 1d). A similar temperature profile was observed for BVDV-1c strain NS155 (*n* = 4), with elevated temperatures on Day 8 and Day 9, with only Day 8 being statistically significant (*p* < 0.05, Figure 1d). Although there was a suggestion of an elevated mean temperature on Day 9 post-infection for the cattle (*n* = 3) infected with VR1112 this was not statistically significant (Figure 1e). The rectal temperatures of the contact animals (*n* = 2) did not exceed 39.0 °C during the experiment (Figure 1f). No statistical comparisons were undertaken for this group due the low animal numbers.

### 2.3. Detection of Virus Post-Infection

The course of the BVDV-1c nasal shedding by the trial cattle was assessed by testing extracts from nasal swabs collected from Day 0 to Day 14 and serum samples collected on Day 7 and Day 14 post-infection with a BVDV-1 specific quantitative real-time (qPCR). A summary of these results expressed as the threshold cycle (Ct) is shown in Table 1. Overall, the distribution of positive nasal swabs was variable between and within the groups of cattle infected with the different strains of BVDV-1c. The earliest that virus was detected in nasal swabs was Day 2 post-infection for animals infected with PI506 (1 of 6 animals), Trangie (1 of 4 animals) and AO554 (2 of 4 animals). One nasal swab from an animal infected with strain PI506 tested positive at Day 14 post infection. 

The BVDV-1c strain AO554 was the virus most consistently detected in the nasal swabs with all infected animals reacting with the qPCR at Day 6 and Day 7 post infection, and three of the four animals also reacted in the qPCR on Day 8 (Table 1). The serum samples collected from animals infected with AO554 at Day 7 post-infection also reacted and were deemed to be positive for BVDV-1c (Table 1).

With respect to the other groups, BVDV-1c was consistently detected in nasal swabs collected on Day 5, Day 6 and Day 7 post-infection from two of the six animals infected with BVDV-1c strain PI506 (Table 1). One of the animals in this group tested positive (nasal swabs) from Day 3 to Day 7 post-infection and the swabs from Day 13 and Day 14 were also positive.

For cattle infected with BVDV-1c strain Trangie, one of the four animals tested positive on Day 2 post-infection, while all the other samples were negative throughout the sampling period (Table 1).

Of the animals infected with BVDV-1c strain NS155, two animals had positive nasal swabs. One of these animals was positive on Day 6. The strain NS155 was detected sporadically between Day 7 and 13 in samples from animal 2680. The serum samples from Day 7 and Day 14 from this animal were both reactive with the qPCR assay (Table 1).

Two animals infected with BVDV-1c strain VR1112 tested positive for the virus in a sporadic manner. The remaining animals did not return any positive results (Table 1). None of the sample extracts from the two contact animals reacted with the qPCR during the sampling period (Table 1).

BVDV-1c was not detected via qPCR in the nasal swab or serum samples collected from all animals on Day 21, Day 28, Day 42 and Day 55 post-infection and were deemed to be negative (data not shown).

### 2.4. Isolation of BVDV-1c from Nasal Swabs

Attempts were made to isolate the BVDV-1c strains from the nasal swabs collected at Day 7 post-infection from selected animals. No BVDV-1c was detected in any of the culture supernatants by qPCR after three passages of Animal 2684 (PI506 infected and tested positive from Day 2 to Day 7), Animal 2686 (Trangie infected), Animal 2666 (AO554 infected) or Animal 2669 (AO554 infected).

### 2.5. Haematological Analyses of Cattle Infected with BVDV-1 Strains

**Figure 2:**
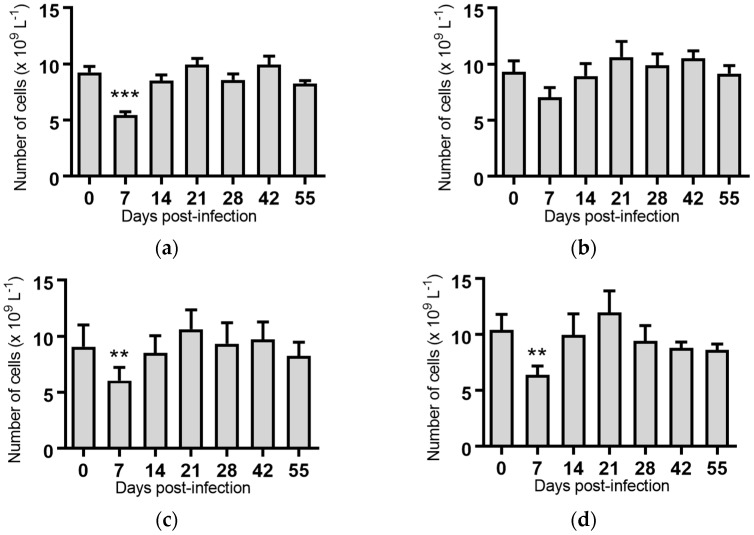

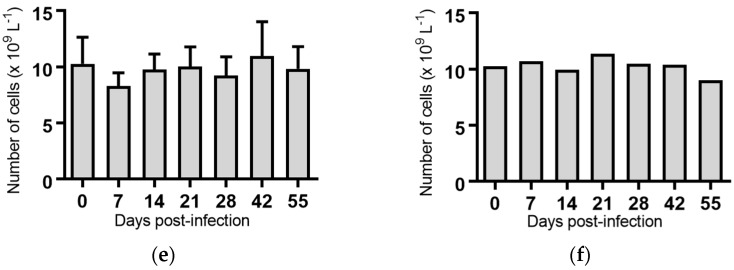
Concentration of white blood cells in blood samples from groups of cattle infected with different strains of bovine viral diarrhoea virus 1 genotype 1c (BVDV-1c). Each column shows the mean concentration of white blood cells for each group and the standard error of the mean. (**a**) Animals infected with BVDV-1c strain PI506 (*n* = 6); (**b**) Animals infected with BVDV-1c strain Trangie (*n* = 4); (**c**) Animals infected with BVDV-1c strain AO554 (*n* = 4); (**d**) Animals infected with BVDV-1c strain NS155 (*n* = 4); (**e**) Animals infected with BVDV-1c strain VR1112 (*n* = 3); (**f**) Uninfected animals (*n* = 2). Mean values which differ significantly from the Day 0 samples for each group are indicated, ** where *p* < 0.01 or *** where *p* < 0.001.

White blood cells: Infection of cattle with BVDV-1c strains PI506, AO554 and NS155 all resulted in a significant depletion of the average white blood cell (WBC) counts at Day 7 post-infection (Figure 2). A trend towards a similar depletion at Day 7 was evident for strains Trangie and VR1112 although this was not statistically significant (Figure 2). At Day 14 post-infection the levels of WBC had returned to similar levels to those observed on the day of infection for all animals (Figure 2).

**Figure 3:**
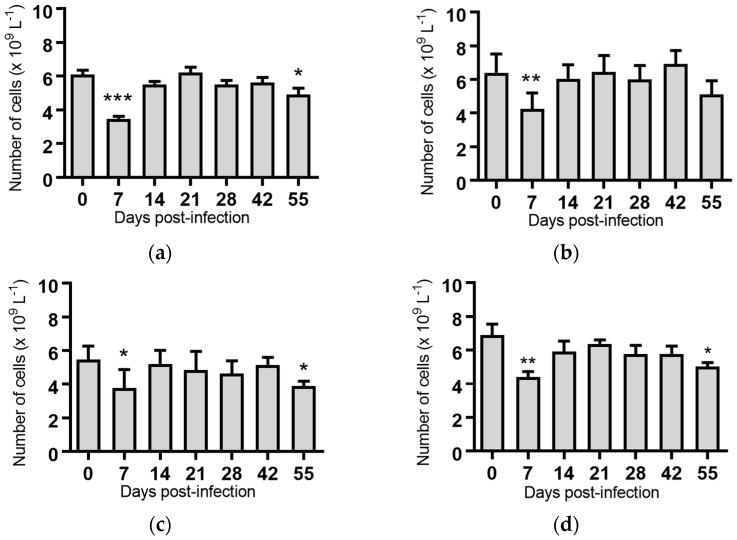

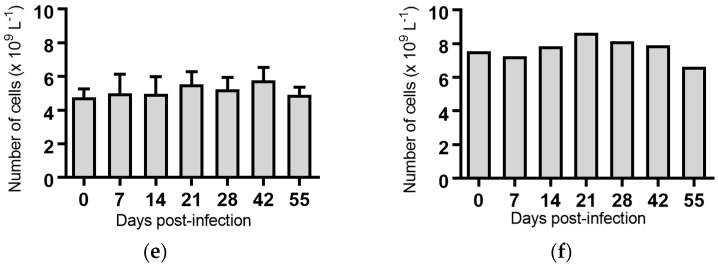
Concentration of lymphocytes in blood samples from groups of cattle infected with different strains of bovine viral diarrhoea virus 1 genotype 1c (BVDV-1c). Each column shows the mean concentration of lymphocytes for each group and the standard error of the mean. (**a**) Animals infected with BVDV-1c strain PI506 (*n* = 6); (**b**) Animals infected with BVDV-1c strain Trangie (*n* = 4); (**c**) Animals infected with BVDV-1c strain AO554 (*n* = 4); (**d**) Animals infected with BVDV-1c strain NS155 (*n* = 4); (**e**) Animals infected with BVDV-1c strain VR1112 (*n* = 3); (**f**) Uninfected animals (*n* = 2). Mean values which differ significantly from the Day 0 samples for each group are indicated, * where *p* < 0.05, ** where *p* < 0.01 or *** where *p* < 0.001.

Lymphocytes: Significant reductions in the mean lymphocyte concentrations were observed for the groups of cattle infected with BVDV-1c strain PI506 (*p* < 0.001, Figure 3a), strain Trangie (*p* < 0.01, Figure 3b), strain AO554 (*p* < 0.05, Figure 3c) and strain NS155 (*p* < 0.01, Figure 3d) on Day 7 post-infection. By Day 14 post-infection the lymphocyte numbers had returned to concentrations similar to those at the start of the trial.

A recurrence of the significant reduction in the concentration of lymphocytes was detected on Day 55 post-infection in cattle infected with strains PI506 (*p* < 0.05, Figure 3a), AO554 (*p* < 0.05, Figure 3c) and NS155 (*p* < 0.05, Figure 3d). While the concentration of lymphocytes appeared to be reduced for cattle infected with strain Trangie on Day 55, this reduction was not statistically significant (Figure 3b). No differences in lymphocytes were evident for the animals infected with strain VR1112 (Figure 3e) or the uninfected animals (Figure 3f).

**Figure 4:**
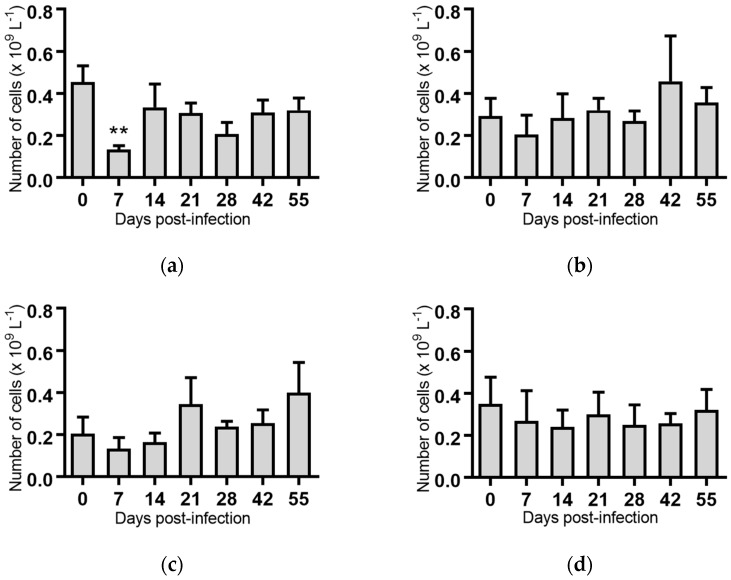

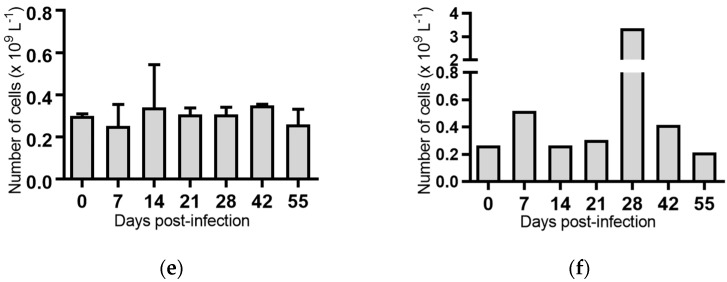
Concentration of monocytes in blood samples from groups of cattle infected with different strains of bovine viral diarrhoea virus 1 genotype 1c (BVDV-1c). Each column shows the mean concentration of monocytes for each group and the standard error of the mean. (**a**) Animals infected with BVDV-1c strain PI506 (*n* = 6); (**b**) Animals infected with BVDV-1c strain Trangie (*n* = 4); (**c**) Animals infected with BVDV-1c strain AO554 (*n* = 4); (**d**) Animals infected with BVDV-1c strain NS155 (*n* = 4); (**e**) Animals infected with BVDV-1c strain VR1112 (*n* = 3); (**f**) Uninfected animals (*n* = 2). Mean values which differ significantly from the Day 0 samples for each group are indicated, ** where *p* < 0.01.

Monocytes: Seven days post-infection, animals infected with BVDV-1c strain PI506 had significantly reduced concentration of monocytes compared to the pre-infection sample (*p* < 0.01, Figure 4a). No significant changes in the numbers of monocytes were detected for any of the animals infected with the remaining BVDV-1c strains or the contact animals (Figure 4).

**Figure 5:**
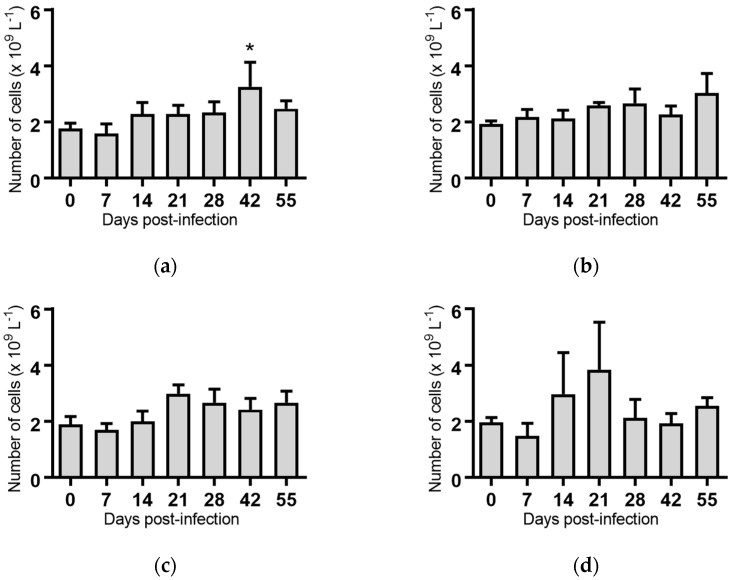

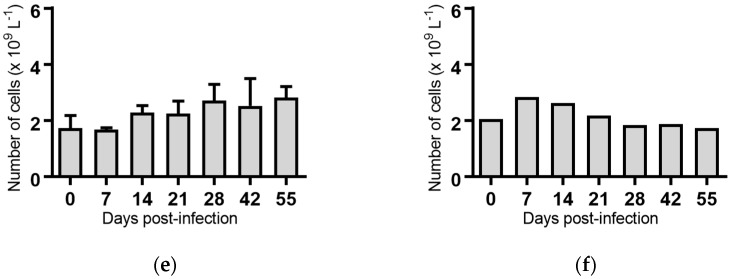
Concentration of neutrophils in blood samples from groups of cattle infected with different strains of bovine viral diarrhoea virus 1 genotype 1c (BVDV-1c). Each column shows the mean concentration of neutrophils for each group and the standard error of the mean. (**a**) Animals infected with BVDV-1c strain PI506 (*n* = 6); (**b**) Animals infected with BVDV-1c strain Trangie (*n* = 4); (**c**) Animals infected with BVDV-1c strain AO554 (*n* = 4); (**d**) Animals infected with BVDV-1c strain NS155 (*n* = 4); (**e**) Animals infected with BVDV-1c strain VR1112 (*n* = 3); (**f**) Uninfected animals (*n* = 2). Mean values which differed significantly from the Day 0 samples are indicated * where *p* < 0.05.

Neutrophils: No significant loss of neutrophils was detected in any of the animals in the trial (Figure 5). On Day 42 post-infection, the group of animals infected with BVDV-1c strain PI506 had a significantly higher concentration of neutrophils compared to the Day 0 samples (Figure 5).

**Figure 6:**
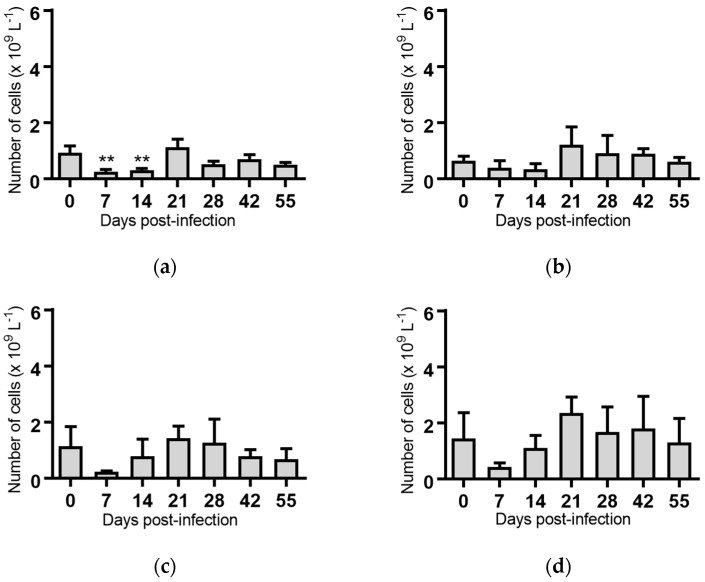

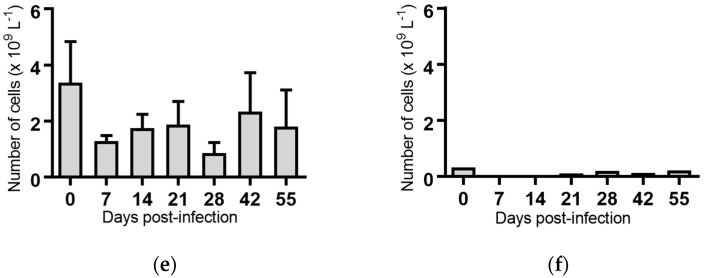
Concentration of eosinophils in blood samples from groups of cattle infected with different strains of bovine viral diarrhoea virus 1 subgenotype 1c (BVDV-1c). Each column shows the mean concentration of eosinophils for each group and the standard error of the mean. (**a**) Animals infected with BVDV-1c strain PI506 (*n* = 6); (**b**) Animals infected with BVDV-1c strain Trangie (*n* = 4); (**c**) Animals infected with BVDV-1c strain AO554 (*n* = 4); (**d**) Animals infected with BVDV-1c strain NS155 (*n* = 4); (**e**) Animals infected with BVDV-1c strain VR1112 (*n* = 3); (**f**) Uninfected animals (*n* = 2). Mean values which differed significantly from the Day 0 samples for strain PI506 are indicated ** where *p* < 0.01.

Eosinophils: The group of animals infected with BVDV-1c strain PI506 had significantly reduced eosinophil concentrations on Day 7 and Day 14 post-infection compared to Day 0 (Figure 6a). None of the blood samples collected from animals infected with the remaining BVDV-1c strains had any significant variation in the number of eosinophils for the duration of the trial. For the remaining groups infected with BVDV-1c strain Trangie (Figure 6b), BVDV-1c strain AO554 (Figure 6c), BVDV-1c strain NS155 (Figure 6d) and BVDV-1c strain VR1112 (Figure 6e) there was a non-significant trend towards a reduced number of eosinophils on Day 7 post-infection. The uninfected control animals consistently had the lowest concentration of eosinophils on all sampling days (Figure 6f).

**Figure 7:**
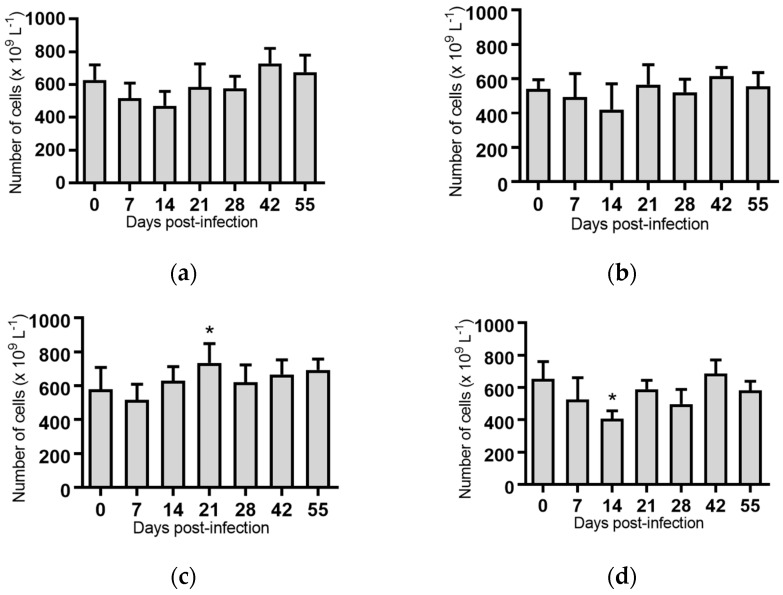

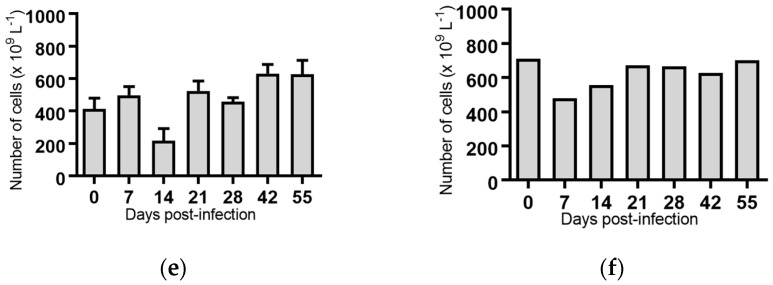
Concentration of platelets in groups of cattle infected with different strains of bovine viral diarrhoea virus 1 subgenotype 1c (BVDV-1c). Each column shows the mean concentration of platelets for each group and the standard error of the mean on each day post-infection. (**a**) Animal infected with BVDV-1c strain PI506 (*n* = 6); (**b**) Animal infected with BVDV-1c strain Trangie (*n* = 4); (**c**) Animal infected with BVDV-1c strain AO554 (*n* = 4); (**d**) Animal infected with BVDV-1c strain NS155 (*n* = 4); (**e**) Animal infected with BVDV-1c strain VR1112 (*n* = 3); (**f**) Uninfected animals (*n* = 2). Mean values which differed significantly from the Day 0 samples for specific groups are indicated * where *p* < 0.05.

Platelets: Overall, the concentration of platelets in infected animals were reduced for most BVDV-1c strains on Day 7 and/or Day 14 post infection compared to Day 0. The only significant reduction was for the animals infected with BVDV-1c strain NS155 on Day 14 (*p* < 0.05, Figure 7d). A reduction in platelet numbers was also apparent on Day 14 for animals infected with strain PI506, Trangie and VR1112, but these differences were not statistically significant (Figure 7a,b,e). In contrast to other infected groups, the concentration of platelets in cattle infected with BVDV-1c strain AO554 appeared stable for the duration of the experiment, apart from a significant increase on Day 21 post-infection (*p* < 0.05, Figure 7c).

### 2.6. Serological Analyses

All trial animals were monitored for the development of BVDV-1 specific antibodies throughout the course of the experiment. Virus specific antibody was first detected on Day 14 post-infection with 10 of the 21 infected animals testing positive. However, none of the animals infected with strains Trangie or AO554 had detectable antibodies by this time point. By Day 21 post-infection, 18 of the 21 animals had detectable BVDV-1 specific antibody (Table 2). All the BVDV-1c challenged animals had detectable BVDV-1 antibodies by Day 28 post-infection (Table 2). One of the animals infected with strain AO554 was positive on Day 14 but subsequently tested negative on Day 21 (Table 2). No BVDV-1 antibody was detected in the serum samples from either of the two contact animals at any of the sampling time points (Table 2).

## 3. Discussion

Several studies have explored the potential links between the subgenotypes and antigenic variation through cross neutralisation studies [3,7,8]. Clearly, knowledge of any such relationship is important as it would facilitate the selection of vaccine components to match the circulating BVDV-1 subgenotypes, while also enabling the ongoing monitoring of field strains to detect any change in the dominant subgenotype.

The importance of the BVDV-1 subgenotypes with respect to the in vivo biology has received minimal attention and more research is required to define commonalities and divergences between each group such as virulence. Several of the parameters measured in this study showed similar effects of the BVDV-1c strains on their bovine hosts. These commonalities were not unexpected as the strains used in this study were all BVDV-1 subgenotype 1c. Currently, there are no specific criteria proposed to evaluate BVDV-1 virulence, however clinical signs (respiratory and/or digestive), biphasic pyrexia, biphasic leukopenia and thrombocytopenia have been reported for BVDV-1 subgenotypes 1a, 1b, 1d, 1e and 1k from various countries [9,10,11,12,13]. In the current study, no respiratory or digestive clinical signs were observed in the BVDV-1c inoculated cattle. 

While a significant pyrexia was identified in cattle infected with four of the five BVDV-1 strains, however, there was no evidence of a biphasic pyrexia (Figure 1). Four of the five BVDV-1 infected groups exhibited significant leukopenia at Day 7 post-infection, three of which were biphasic (Figure 3). Only the group infected with VR1112 did not have detectable leukopenia. As VR1112 was the only cytopathic BVDV-1 strain included in the current study, further research is required to determine why this was the case. With respect to thrombocytopenia, only the cattle infected with strain NS155 had a significant loss of platelets that was detected 14 days after infection (Figure 7d). Collectively these data suggest the BVDV-1 subgenotypes 1c evaluated in this study have low virulence in transiently infected animals under the experimental conditions utilised. 

One additional parameter which is commonly considered in the assessment of viral virulence is transmission capacity [14]. There is general agreement that there is either no or limited horizontal transmission of BVDV-1 between transiently infected cattle [15,16,17]. Sarrazin et al. [18] concluded that the field strains of BVDV-1 subgenotype 1a and 1b evaluated in their study were unlikely to play an important role in transmission. The detection of BVDV-1c in the nasal swabs of infected cattle the current study was sporadic and where detected the results suggested low quantities of virus (Table 1). The range of Ct values from nasal swabs and serum samples in the current study were 31.6 to 39.0 and 32.0 to 39.0 respectively (Table 1). Previous studies have evaluated the use of qPCR to differentiate persistently and transiently infected animals. Hanon et al. [19] estimated that a Ct value below 28.89 from a blood sample would identify all persistently infected animals, although this value was likely to misclassify some transiently infected animals as being persistently infected. While Hay et al. [20] estimated that a Ct value below 33 from serum was indicative of an animal being persistently infected with BVDV-1. The results of the current study, suggest a Ct value of 33 is too high for the differentiation of transiently and persistently infected animals based on a single sample. Noting that both prior studies utilised field samples for these estimates, thus direct comparison to the current study requires caution. The use of these estimates, would also require sample preparation and analyses to be comparable, particularly volume of sample extracted and subsequently used in the qPCR assay.

Associated with these results, the two uninfected control animals included in the study did not test positive for BVDV-1 or seroconvert to BVDV-1 over the course of the experiment. Unchallenged animals could only be included in one of the containment rooms of the study for logistical reasons. Consequently, there were no uninfected animals penned with the animals infected with BVDV-1c strain AO554 or strain PI506, the viruses most consistently detected in nasal swabs and in the highest quantities (Table 1). Collectively, these results suggest that minimal amounts of the challenge viruses were present in the nasal secretions of the infected animals and as a result the risk of virus transmission to other animals was very low for these BVDV-1c strains. Evans et al. [21] recently reported the absence of horizontal transmission from sheep experimentally infected with an Australian strain of BVDV-1 subgenotype 1c to sentinel sheep. The possibility of animals transiently infected with the BVDV-1c strains used in these studies producing and shedding sufficient quantities of virus to facilitate transmission if subjected to stressful conditions cannot be excluded. It has recently been demonstrated that the BVDV-1 strain H0916 (subgenotype 1a) was only transmitted to sentinel animals when the infected animals were immunosuppressed with dexamethasone [22]. If the low risk if transmission from transiently infected animals extends to all BVDV-1 subgenotype 1c it could have important implications in the implementation of effective BVDV-1 control plans with persistently infected animals as the sole source of virus [3,23,24,25]. Further research is required to determine if any BVDV-1 subgenotype 1c strains replicate sufficiently in the nasal epithelia at levels to facilitate transmission to susceptible sentinel animals. The BVDV-1c strains PI506 and AO554 would be excellent candidate viruses for these studies.

While the detection of virus in nasal swab and serum samples was sporadic, the cattle were clearly infected as demonstrated by the serological analyses. Infected animals started to seroconvert by Day 14 post-infection with 10 of the 22 infected animals testing positive for BVDV-1 specific antibody. The number of positive animals increased by Day 21, with all infected animals being antibody positive by Day 28. These data are consistent with other BVDV-1 infection studies [17,26]. There did not appear to be anything specific to the BVDV-1c strains used in this study in relation to the serological data with animals from all groups becoming seropositive in a fourteen day period and all animals being seropositive by Day 28.

The impacts of some BVDV-1c strains on cattle in the current study may have been under-estimated due to the smaller number of cattle in each group, particularly for strain VR1112 where one animal was withdrawn from the experiment immediately prior to commencement of the trial for ethical reasons (lameness). The number of cattle in this study was constrained by the capacity of the facility and need to evaluate the properties of several BVDV-1 subgenotype 1c strains. Another potential limitation of the current study was the viral inoculums used were quantified using RT-qPCR of the final cell culture supernatants. While previous studies have demonstrated correlations between RT-qPCR results and measures of in vitro infectivity such as plaque forming units and/or 50% cell culture infectious dose (TCID50) for other viruses, such relationships have not been reported for BVDV-1 as yet [27,28,29,30]. Future studies which aim to directly compare the in vivo properties of the BVDV-1c strains used in the study would need to establish the relationship between RT-qPCR results and measures of in vitro infectivity to enable the standardisation of the challenge doses.

Future studies will be required to better understand the relationships between the BVDV-1 subgenotypes and virulence. Strong et al. [22] also identified that the challenge dose can influence the clinical outcomes of cattle challenged with BVDV-1a. Data was also reported which suggested an influence of calf age on clinical outcome. As a consequence, future studies aiming to characterise the interactions of BVDV-1 and its bovine host should aim to do so under a standardised challenge system, including route of infection, challenge dose (where possible multiple doses) and age of animals. It is imperative that the subgenotype of the BVDV-1 isolate(s) used also be included.

While the current study has focused on the respiratory component of the BVDV-1c infection, it is well accepted the virus can have profound impacts on the reproductive capacity of individual animals and cattle herds overall. The BVDV-1c isolate Trangie was used in several earlier studies that reported the capacity of this virus to significantly impair bovine reproductive function [31,32,33,34]. The capacity of specific BVDV-1 strains and/or subgenotype groups to cause both respiratory and reproductive disease are yet to be investigated, and may be required to fully understand this important cattle pathogen.

This study is the first to characterise the in vivo properties of BVDV-1 strains confirmed as belonging to the subgenotype 1c. Interestingly, the overall impacts of the infection of the different strains on the infected cattle were in general similar to those reported for other BVDV-1 subgenotypes with transient pyrexia, leukopenia, and quantities of virus in nasal swabs which are unlikely to facilitate horizontal transmission [9,10,11,12,13,22]. Of the BVDV-1c strains examined in this study PI506 had the most consistent impact on the experimentally infected cattle and is a strong candidate for use in cattle studies to further define the in vivo properties of the subgenotype 1c, including direct comparisons to strains of other subgenotypes.

## 4. Materials and Methods

### 4.1. Animal Ethics

All experimental procedures involving animals were reviewed and approved by The University of Queensland Animal Ethics Committee, Approval number QAAFI/399/13/MLA.

### 4.2. Cattle Infection Trial

The BVDV-1c isolates used in the cattle trial are described in Table 3. Viral inoculums were prepared by adding 100 μL of primary stock of each BVDV-1c strain to culture medium of subconfluent monolayers of MDBK cells in tissue culture flasks (25 cm^2^) and incubated at 37 °C in a 5% CO_2_ atmosphere for seven days. The supernatants were clarified at 5000 g, aliquoted and stored at −80 °C until required. As the aims of this study did not include intergroup statistical comparisons, the viral supernatants were used as harvested to inoculate cattle at the maximum possible titre. Cattle were sourced by Veterinary Health Research Ltd. Pty (Armidale, NSW, Australia). The animals were Black Angus and 6 to 9 months of age. Prior to enrolment into the study, cattle were tested multiple times and confirmed negative for serological evidence of prior BVDV-1 exposure/infection. ELISAs were performed using the BIO K284 ELISA, as described by the manufacturer (Bio-X Diagnostics, Jemelle, Belgium). 

Seven days prior to commencement of the trial, cattle (*n* = 24) were moved into the Large Animal PC2 facility at the Queensland Animal Science Precinct (Gatton, QLD, Australia). The cattle were randomly assigned to one of four pens (4 × 6) with two pens per room (Table 4). The rooms are operated independently, including separate air handling systems. To minimise the risk of virus transmission between rooms, separate teams of staff were used to maintain/care for and collect samples from the animals in each room daily.

On Day 0, the rectal temperature for each animal was recorded and two blood samples collected via the jugular vein. The cattle groups were inoculated with one of the BVDV-1 viral strains as shown in Table 4. Briefly, the animal was restrained with the nose elevated and the viral inoculum (1 mL) was slowly dripped into each nostril. The nose was held in this position for 15 to 20 s and the animal then released. Two animals in Room 2 were not inoculated with virus.

Clinical assessments: Cattle were monitored from Day 1 to Day 14 post-infection for clinical signs in respect to nasal discharge, coughing, behavior/demeanour and loss of appetite (feed residue). 

Temperature: The rectal temperatures for each animal was recorded from Day 1 to Day 14. The expected rectal temperature of healthy cattle was 38.5 °C [36].

### 4.3. Virus Detection in Nasal Swabs and Sera

Nasal swabs were collected from Day 0 to Day 14, Day 21, Day 28, Day 42, Day 56 and Day 60. Nasal swabs were immediately placed on ice for transport back to the laboratory for storage at 4 °C until required. Nasal swabs were immersed in 500 μL of PBS containing 5 × Antibiotic-Antimycotic (Thermofisher Scientific, Waltham, MA, USA) and gently agitated. The swab was subsequently removed and discarded. A 200 μL aliquot of this resuspension was used for total nucleic acid extraction using the DNeasy Blood & Tissue Kit (QIAGEN, Hilden, Germany) as described by the manufacturer, except for the exclusion of RNAse A. Total nucleic acid extracts were also prepared from aliquots (200 μL) of cattle serum samples, collected as described below, using the same methodology. Sample extracts prepared from nasal swabs and sera were analysed by qPCR for the presence of BVDV-1 RNA as previously described [37]. Samples yielding a Ct value ≥ 40 were deemed to be negative for BVDV-1.

### 4.4. Virus Isolation from Nasal Swabs

An aliquot of the resuspended nasal swab from Day 7 post-infection from selected animals were utilised for virus isolation. Aliquots, 10 μL and 100 μL of the nasal swab resuspension diluted 1:10 with PBS were added directly to culture medium of subconfluent monolayers of MDBK cells in 6 well plates and incubated at 37 °C in a 5% CO_2_ atmosphere for seven days. The monolayers were freeze/thawed once and a 100 μL aliquot of the culture supernatant added to new subconfluent monolayers of MDBK cells in 6 well plates and incubated at 37 °C in a 5% CO_2_ for seven days. This process was repeated three times. Total nucleic acids were extracted from a 200 μL aliquot of each culture supernatant and tested using qPCR for the presence of BVDV-1 as described previously.

### 4.5. Serology and Haematology

Blood sampling: Blood for serum harvesting (6 mL BD Vacutainers™, BD Biosciences, Franklin Lakes, NJ, USA) and blood cell count analyses (6 mL EDTA BD Vacutainers™, BD Scientific, Franklin Lakes, NJ, USA) were collected on Day 0, Day 7, Day 14, Day 21, Day 28, Day 42, Day 56 and Day 60 post infection.

Serum samples were tested for the presence of BVDV-1 specific antibodies using the BIO K284 ELISA, as described by the manufacturer (Bio-X Diagnostics, Jemelle, Belgium). The level of virus specific IgG in each serum sample was assigned to one of six arbitrary categories, negative (−) or positive (+, ++, +++, ++++, or +++++) according to the manufacturer’s instructions. 

Whole blood samples were submitted to the Veterinary Science Diagnostic Services (School of Veterinary Science, University of Queensland, Gatton, QLD, Australia) for analyses of the cell populations using standard blood smearing and cell counting methodologies. 

### 4.6. Statistical Analyses

Data generated from the animal trial were analysed using a one-way analysis of variance (ANOVA) with Dunnett’s multiple comparisons test and statistical significance attributed where *p* < 0.05 within Graphpad PRISM™ (Version 7.03, GraphPad Software, Inc., La Jolla, CA, USA).

## Figures and Tables

**Table 1:** Detection of bovine viral diarrhoea virus 1 subgenotype 1c in extracts from cattle samples using quantitative real time PCR (qPCR). Nasal swabs (N) and serum (S) samples were analysed using qPCR. Reactive samples were deemed positive for BVDV-1c with the cycle threshold value shown (shaded cells). Samples which did not react (>40) with the qPCR were deemed to be negative (−) for the virus.

		Days Post Infection ^1^														
		2	3	4	5	6	7	7	8	9	10	11	12	13	14	14
Virus	Animal ID	N	N	N	N	N	N	S	N	N	N	N	N	N	N	S
PI506 ^2^	2661	−	−	−	−	−	−	−	−	−	−	−	−	−	−	−
	2664	−	35.6	−	−	−	−	−	−	−	−	−	−	−	−	−
	2665	−	35.4	−	−	−	−	−	−	−	−	−	−	−	−	−
	2676	−	−	−	37.8	36.6	36.7	36.0	37.1	−	−	−	−	−	−	−
	2682	−	−	−	−	−	−	−	37.1	−	−	−	−	−	−	−
	2684 ^4^	31.7	32.8	36.7	36.5	34.8	32.8	34.7	−	−	−	−	−	36.5	36.5	−
Trangie ^2^	2675	−	−	−	−	−	−	−	−	−	−	−	−	−	−	−
	2677	−	−	−	−	−	−	−	−	−	−	−	−	−	−	−
	2685	−	−	−	−	−	−	−	−	−	−	−	−	−	−	−
	2686 ^4^	39.0	−	−	−	−	−	−	−	−	−	−	−	−	−	−
AO554 ^3^	2666 ^4^	35.3	34.3	−	−	33.6	32.0	32.0	36.3	−	−	−	−	−	−	−
	2668	−	−	34.8	39.5	33.8	32.4	35.7	−	−	37.3	−	−	−	−	−
	2669 ^4^	−	36.6	37.1	−	32.0	31.6	33.7	32.6	37.5	−	−	−	−	−	−
	2673	37.0	−	−	−	37.3	39.8	35.9	37.4	−	−	−	−	−	−	−
NS155 ^3^	2667	−	−	−	−	−	−	−	−	−	−	−	−	−	−	−
	2672	−	−	−	−	−	−	−	−	−	−	−	−	−	−	−
	2674	−	−	−	−	38.4	−	−	−	−	−	−	−	−	−	−
	2680	−	−	−	−	−	−	34.3	36.5	−	−	−	−	34.6	−	39.0
VR1112 ^3^	2662	−	−	−	−	−	−	−	−	−	−	−	−	−	−	−
	2678	−	36.8	38.8	−	−	−	−	−	−	−	−	−	−	−	−
	2683	−	−	−	−	−	−	−	−	−	−	−	−	39.5	−	−
Contact ^2^	2663	−	−	−	−	−	−	−	−	−	−	−	−	−	−	−
	2681	−	−	−	−	−	−	−	−	−	−	−	−	−	−	−

^1^ Samples collected on Day 0 and Day 1 were non-reactive with the qPCR assay. ^2^ Animals housed together in Containment Room 2. ^3^ Animals housed together in Containment Room 1. ^4^ Virus isolation experiments using the Day 7 nasal swabs taken from selected animals did not recover any detectable virus.

**Table 2:** Serological responses (IgG) of cattle challenged with one of five strains of bovine viral diarrhoea virus 1 genotype 1c. The level of virus specific IgG were determined using a commercial ELISA and assigned to one of six arbitrary categories, negative (−) or positive (+, ++, +++, ++++, or +++++).

Strain	Animal ID	Days Post-Infection						
		0	7	14	21	28	35	55
PI506	2661	−	−	−	+	++	+++	++
	2664	−	−	+	+++	++++	+++++	++++
	2665	−	−	+	+++	++++	++++	+++
	2676	−	−	−	++	+++	++++	++++
	2682	−	−	−	++	++	++++	++++
	2684	−	−	−	+++	+++	++++	++++
Trangie	2675	−	−	−	+++	++++	++++	+++
	2677	−	−	−	++	+++	+++	+++
	2685	−	−	−	+++	+++	++++	+++++
	2686	−	−	++	+++	+++++	+++++	+++++
AO554	2666	−	−	++	+++	++++	+++++	++++
	2668	−	−	+++	−	+++++	+++++	+++++
	2669	−	−	+++	++++	+++++	+++++	+++++
	2673	−	−	−	++	++++	+++++	++++
NS155	2667	−	−	+++	+++	+++++	+++++	++++
	2672	−	−	+	+	+++++	+++++	++++
	2674	−	−	−	−	++++	++++	++++
	2680	−	−	+	++++	+++++	+++++	+++++
VR1112	2662	−	−	−	−	++	++	++
	2678	−	−	+++	+++	+++++	+++++	+++++
	2683	−	−	−	+	+	++	++
Contact	2663	−	−	−	−	−	−	−
	2681	−	−	−	−	−	−	−

**Table 3:** Properties of the bovine viral diarrhea virus 1 isolates used to study.

Isolate	Biotype	Passage	qPCR ^1^	Source
PI506	Non-cytopathic	P3	19	Isolated from a persistently infected animal
Trangie ^2^	Non-cytopathic	P3	17	Dr Peter Kirkland (DPI) [35]
AO554	Non-cytopathic	P3	18	Isolated from a persistently infected animal
VR1112 ^2^	Cytopathic	P4	17, (1 × 10^4.6^ TCID50 mL^−1^)	Dr Jan Smith (JCU)
NS155	Non-cytopathic	P3	22	Isolated from a feedlot animal, treated for bovine respiratory disease

^1^ Real-time PCR threshold cycle value of virus inoculum. The TCID50 of the cytopathic strain is shown in parenthesis. ^2^ Isolate was positive for mycoplasma and was treated with Plasmocin (Sigma-Aldrich, St Louis, MO, USA) according to the manufacturer’s instructions until PCR test was negative.

**Table 4:** Experimental layout utilised in the bovine viral diarrhea virus 1 (BVDV-1) infection study.

Room	Pen Number	Strain	Number of Cattle
1	1.1	AO554	4
1	1.1	Trangie	2
1	1.2	Trangie	2
1	1.2	VR1112	3 ^1^
2	2.3	PI506	6
2	2.4	NS155	4
2	2.4	No virus	2

^1^ One animal was withdrawn for ethical reasons prior to inoculation.

## References

[1] Yesilbag K., Alpay G., Becher P. (2017). Variability and global distribution of subgenotypes of bovine viral diarrhea virus. Viruses.

[2] Mahony T.J., McCarthy F.M., Gravel J.L., Corney B., Young P.L., Vilcek S. (2005). Genetic analysis of bovine viral diarrhoea viruses from Australia. Vet. Microbiol..

[3] Ridpath J.F., Fulton R.W., Kirkland P.D., Neill J.D. (2010). Prevalence and antigenic differences observed between bovine viral diarrhea virus subgenotypes isolated from cattle in Australia and feedlots in the southwestern United States. J. Vet. Diagn. Investig..

[4] Fulton R.W., Ridpath J.F., Saliki J.T., Briggs R.E., Confer A.W., Burge L.J., Purdy C.W., Loan R.W., Duff G.C., Payton M.E. (2002). Bovine viral diarrhea virus (BVDV) 1b: Predominant BVDV subtype in calves with respiratory disease. Can. J. Vet. Res..

[5] Wernike K., Schirrmeier H., Strebelow H.G., Beer M. (2017). Eradication of bovine viral diarrhea virus in Germany-diversity of subtypes and detection of live-vaccine viruses. Vet. Microbiol..

[6] Ridpath J.F. (2010). Bovine viral diarrhea virus: Global status. Vet. Clin. N. Am. Food Anim. Pract..

[7] Abe Y., Tamura T., Torii S., Wakamori S., Nagai M., Mitsuhashi K., Mine J., Fujimoto Y., Nagashima N., Yoshino F. (2016). Genetic and antigenic characterization of bovine viral diarrhea viruses isolated from cattle in Hokkaido, Japan. J. Vet. Med. Sci..

[8] Nagai M., Ito T., Sugita S., Genno A., Takeuchi K., Ozawa T., Sakoda Y., Nishimori T., Takamura K., Akashi H. (2001). Genomic and serological diversity of bovine viral diarrhea virus in Japan. Arch. Virol..

[9] Galav V., Mishra N., Dubey R., Rajukumar K., Pitale S.S., Shrivastav A.B., Pradhan H.K. (2007). Pathogenicity of an Indian isolate of bovine viral diarrhea virus 1b in experimentally infected calves. Res. Vet. Sci..

[10] Glotov A.G., Glotova T.I., Koteneva S.V., Semenova O.V., Sergeev A.A., Titova K.A., Morozova A.A., Sergeev A.A. (2016). Virulent properties of russian bovine viral diarrhea virus strains in experimentally infected calves. Scientifica (Cairo).

[11] Larska M., Polak M.P., Riitho V., Strong R., Belak S., Alenius S., Uttenthal A., Liu L. (2012). Kinetics of single and dual infection of calves with an Asian atypical bovine pestivirus and a highly virulent strain of bovine viral diarrhoea virus 1. Comp. Immunol. Microbiol. Infect. Dis..

[12] Ridpath J.F., Neill J.D., Peterhans E. (2007). Impact of variation in acute virulence of BVDV1 strains on design of better vaccine efficacy challenge models. Vaccine.

[13] Wang W., Shi X., Tong Q., Wu Y., Xia M.Q., Ji Y., Xue W., Wu H. (2014). A bovine viral diarrhea virus type 1a strain in China: Isolation, identification, and experimental infection in calves. Virol. J..

[14] Houe H. (1999). Epidemiological features and economical importance of bovine virus diarrhoea virus (BVDV) infections. Vet. Microbiol..

[15] Niskanen R., Lindberg A., Larsson B., Alenius S. (2000). Lack of virus transmission from bovine viral diarrhoea virus infected calves to susceptible peers. Acta Vet. Scand..

[16] Niskanen R., Lindberg A., Traven M. (2002). Failure to spread bovine virus diarrhoea virus infection from primarily infected calves despite concurrent infection with bovine coronavirus. Vet. J..

[17] Collins M.E., Heaney J., Thomas C.J., Brownlie J. (2009). Infectivity of pestivirus following persistence of acute infection. Vet. Microbiol..

[18] Sarrazin S., Dewulf J., Mathijs E., Laureyns J., Mostin L., Cay A.B. (2014). Virulence comparison and quantification of horizontal bovine viral diarrhoea virus transmission following experimental infection in calves. Vet. J..

[19] Hanon J.B., Van der Stede Y., Antonissen A., Mullender C., Tignon M., van den Berg T., Caij B. (2014). Distinction between persistent and transient infection in a bovine viral diarrhoea (bvd) control programme: Appropriate interpretation of real-time RT-PCR and antigen-ELISA test results. Transbound Emerg. Dis..

[20] Hay K.E., Ambrose R.C., Morton J.M., Horwood P.F., Gravel J.L., Waldron S., Commins M.A., Fowler E.V., Clements A.C., Barnes T.S. (2016). Effects of exposure to bovine viral diarrhoea virus 1 on risk of bovine respiratory disease in Australian feedlot cattle. Prev. Vet. Med..

[21] Evans C.A., Moffat J.L., Hemmatzadeh F., Cockcroft P.D. (2017). The risk of transmission from sheep experimentally infected with bvdv-1c during the acute phase to bvdv naïve sheep. Small Ruminant Res..

[22] Strong R., La Rocca S.A., Paton D., Bensaude E., Sandvik T., Davis L., Turner J., Drew T., Raue R., Vangeel I. (2015). Viral dose and immunosuppression modulate the progression of acute bvdv-1 infection in calves: Evidence of long term persistence after intra-nasal infection. PLoS ONE.

[23] Ridpath J.F. (2005). Practical significance of heterogeneity among BVDV strains: Impact of biotype and genotype on U.S. control programs. Prev. Vet. Med..

[24] Fulton R.W., Ridpath J.F., Ore S., Confer A.W., Saliki J.T., Burge L.J., Payton M.E. (2005). Bovine viral diarrhoea virus (BVDV) subgenotypes in diagnostic laboratory accessions: Distribution of BVDV1a, 1b, and 2a subgenotypes. Vet. Microbiol..

[25] Lindberg A., Houe H. (2005). Characteristics in the epidemiology of bovine viral diarrhea virus (BVDV) of relevance to control. Prev. Vet. Med..

[26] Fredriksen B., Sandvik T., Loken T., Odegaard S.A. (1999). Level and duration of serum antibodies in cattle infected experimentally and naturally with bovine virus diarrhoea virus. Vet. Rec..

[27] Jonsson N., Gullberg M., Lindberg A.M. (2009). Real-time polymerase chain reaction as a rapid and efficient alternative to estimation of picornavirus titers by tissue culture infectious dose 50% or plaque forming units. Microbiol. Immunol..

[28] Kallesh D.J., Hosamani M., Balamurugan V., Bhanuprakash V., Yadav V., Singh R.K. (2009). Quantitative PCR: A quality control assay for estimation of viable virus content in live attenuated goat pox vaccine. Indian J. Exp. Biol..

[29] Iwami S., Holder B.P., Beauchemin C., Morita S., Tada T., Sato K., Igarashi T., Miura T. (2012). Quantification system for the viral dynamics of a highly pathogenic simian/human immunodeficiency virus based on an in vitro experiment and a mathematical model. Retrovirology.

[30] Gustafsson R.K.L., Engdahl E.E., Fogdell-Hahn A. (2012). Development and validation of a Q-PCR based TCID(50) method for human herpesvirus 6. Virol. J..

[31] McGowan M.R., Kafi M., Kirkland P.D., Kelly R., Bielefeldt-Ohmann H., Occhio M.D., Jillella D. (2003). Studies of the pathogenesis of bovine pestivirus-induced ovarian dysfunction in superovulated dairy cattle. Theriogenology.

[32] McGowan M.R., Kirkland P.D. (1995). Early reproductive loss due to bovine pestivirus infection. Br. Vet. J..

[33] McGowan M.R., Kirkland P.D., Richards S.G., Littlejohns I.R. (1993). Increased reproductive losses in cattle infected with bovine pestivirus around the time of insemination. Vet. Rec..

[34] McGowan M.R., Kirkland P.D., Rodwell B.J., Kerr D.R., Carroll C.L. (1993). A field investigation of the effects of bovine viral diarrhea virus infection around the time of insemination on the reproductive performance of cattle. Theriogenology.

[35] Brown L.M., Papa R.A., Frost M.J., Mackintosh S.G., Gu X., Dixon R.J., Shannon A.D. (2002). A single amino acid is critical for the expression of B-cell epitopes on the helicase domain of the pestivirus NS3 protein. Virus. Res..

[36] Ali T.M. (1994). A Manual for the Primary Animal Health Care Worker.

[37] Horwood P.F., Mahony T.J. (2011). Multiplex real-time RT-PCR detection of three viruses associated with the bovine respiratory disease complex. J. Virol. Methods.

